# Optimal Control of Shigellosis with Cost-Effective Strategies

**DOI:** 10.1155/2020/9732687

**Published:** 2020-08-28

**Authors:** Stephen Edward, Nyimvua Shaban, Eunice Mureithi

**Affiliations:** ^1^Department of Mathematics, University of Dar es Salaam, Box 35062, Dar es Salaam, Tanzania; ^2^Department of Mathematics and Statistics, University of Dodoma, Box 338, Dodoma, Tanzania

## Abstract

In this paper, we apply optimal control theory to the model for shigellosis. It is assumed that education campaign, sanitation, and treatment are the main controls for this disease. The aim is to minimize the number of infections resulting from contact with careers, infectious population, and contaminated environments while keeping the cost of associated controls minimum. We achieve this aim through the application of Pontryagin's Maximum Principle. Numerical simulations are carried out by using both forward and backward in time fourth-order Runge-Kutta schemes. We simulate the model under different strategies to investigate which option could yield the best results. The findings show that the strategy combining all three control efforts (treatment, sanitation, and education campaign) proves to be more beneficial in containing shigellosis than the rest. On the other hand, cost-effectiveness analysis is performed via incremental cost-effectiveness ratio (ICER). The findings from the ICER show that a strategy incorporating all three controls (treatment, sanitation, and education campaign) is the most cost-effective of all strategies considered in the study.

## 1. Introduction

Shigellosis is an acute infection of the intestine caused by bacteria in the genus *Shigella*. There are four species of *Shigella*: S*higella dysenteriae*, *S. flexneri*, *S. boydii*, and *S. sonnei* (also referred to as groups A, B, C, and D, respectively). An estimate of 165 million cases of shigellosis is reported annually worldwide [1, 2]. Illness can range from mild diarrhea to potentially fatal dysentery, depending on *Shigella* species and host factors. Secondary infections are common due to the low infectious dose. Since humans and other primates are the sole natural reservoirs for *Shigella* and a shigellosis vaccine is not available, rigorous human hygiene practices are the cornerstone of prevention of foodborne transmission [3].

The symptoms of shigellosis vary from mild diarrhea lasting a few days to an acute febrile illness that may include nausea, vomiting, tenesmus, and bloody stools. Symptoms begin 1-4 days after infection and typically last 4-7 days; they are usually self-limited and infrequently require hospitalization. Children under five years, the elderly, and immunocompromised are at higher risk of severe illness. Mild cases of shigellosis are often undiagnosed and not treated; asymptomatic infection is also possible. Once infected, individuals are not likely to get infected again with the same species for several years [3].

Shigellosis is mainly transmitted via fecal-oral route. The organism does not persist long term in the environment, but it can survive in foods at ambient or refrigerated temperatures in sufficient quantities to cause illness for the duration of the shelf life of some foods. Person-to-person transmission is also common in this disease [4–6].

Several scholars have studied shigellosis by mathematical models with the main focus of understanding its transmission dynamics (e.g., see [7–11]). Motivated by the work of Edward et al. [11] who studied shigellosis by examining the role played by carriers in its transmission dynamics, we apply optimal control theory to study how the optimal control strategies could be designed to end this disease.

Optimal control is a branch of mathematics which deals with finding optimal ways to control a dynamical system. The theory has been currently used extensively in many fields such as biological sciences, economics, physics, and engineering to mention a few [12]. In mathematical epidemiology, this theory has been a useful tool when it comes to planning how to eliminate or minimize the number of cases at an optimal cost. Several studies have used the optimal control theory to capture intervention strategies, e.g., see [13]. They used the optimal control theory to confirm the significant role played by control measures (education and treatment of water bodies) and the bacteria in the environment in the transmission dynamics as well as reduce the spread of cholera. Reference [14] studied cholera by incorporating two control strategies, namely, education and chlorination. Cost-effectiveness was also carried out, and it was noted that education was the most cost-effective strategy to curtail cholera. Reference [15] developed a cholera epidemiological model which incorporates three types of intervention strategies: vaccination, therapeutic treatment, and water sanitation. Optimal control theory was then applied to seek the cost-effective solution of multiple time-dependent intervention strategies against cholera outbreaks. Reference [16] studied dysentery with optimal control strategies. They applied incremental cost-effectiveness analysis technique to determine the most cost-effective strategy. It was noted that sanitation and education campaign are the most efficient and cost-effective.

Most previous studies of shigellosis did not invest in optimal control strategies, except possibly a study by Berhe et al. [16]. However, their basic model has a few shortcomings that have been addressed by Edward et al. [11]. Therefore, this study focuses on identifying optimal control strategies for the model developed by Edward et al. [11]. We propose and analyze shigellosis optimal control problem that captures three controls, namely, treatment, sanitation, and public health education campaign. With these interventions, individuals are protected from infection. The objective is to find the optimal strategy that minimizes the total number of new infections while keeping the cost associated with the strategy low. Pontryagin's Maximum Principle [17] is used to find the optimal level of effort, which gives the required control of the disease at the cheapest cost. Furthermore, this study investigates which control strategy is the most cost-effective; this is made possible via ICER.

The rest of the paper is organized as follows: Section 2 focuses on the formulation of an optimal control problem and investigating its existence, then deriving the optimality system which characterizes the optimal control using Pontryagin's Maximum Principle. On the other hand, numerical simulation is presented in Section 3.  Section 4 presents a cost-effective analysis of the control strategies, and lastly, Section 5 winds up by giving concluding remarks.

## 2. A Model for Optimal Control Problem

The present study extends the work by Edward et al. [11] which included treatment, public health education campaign, and sanitation as constant control measures. The main difference between the previous work by Edward et al. [11] and the current study is that the present one hinges on application of the optimal control theory. In this case, the constant parameters are treated as time-dependent variables; such a notion allows us to explore how the disease can be optimally controlled using a suitable strategy which is cost-effective. To formulate an optimal control problem, first consider a basic model (1) developed by Edward et al. [11] whose parameters and variables are given in Tables 8 and 9:

(1) $$\begin{array}{c} \frac{dS}{dt}=\Lambda +\omega R-\left(\left(1-\rho \right)\left(\lambda_{h}\left(t\right)+\lambda_{p}\left(t\right)\right)+\mu_{h}\right)S,\\ \frac{dE}{dt}=\left(1-\rho \right)\left(\lambda_{h}\left(t\right)+\lambda_{p}\left(t\right)\right)S-\left(\mu_{h}+\delta \right)E,\\ \frac{dI}{dt}=q\delta E+\left(1-l\right)\alpha C-\left(\mu_{h}+d_{1}+\eta_{1}+\gamma \right)I,\\ \frac{dC}{dt}=\left(1-q\right)\delta E-\left(\mu_{h}+l\eta_{2}+\left(1-l\right)\alpha \right)C,\\ \frac{dR}{dt}=\left(\eta_{1}+\gamma \right)I+l\eta_{2}C-\left(\mu_{h}+\omega \right)R,\\ \frac{dB}{dt}=rB\left(1-\frac{B}{K_{p}}\right)+\left(1-\rho \right)\varepsilon_{1}I+\left(1-\rho \right)\varepsilon_{2}C-\left(\mu_{b}+\sigma \right)B, \end{array}$$

where

(2) $$\begin{array}{c} \lambda_{h}\left(t\right)=\beta_{1}I+\beta_{2}C,\\ \lambda_{p}\left(t\right)=\frac{\phi B}{K+B}, \end{array}$$

with initial conditions *S*(0) > 0; *E*(0) > 0; *I*(0) > 0; *C*(0) > 0; *R*(0) > 0; and *B*(0) > 0.

Next, it is assumed that effective treatment of shigellosis patients is imperative in reducing the spread of the disease. If shigellosis patients are left untreated for long, situations may be fatal as in most cases, clients die due to dehydration. Therefore, we assume that infectious individuals are treated at the rate *u*_1_(*t*) and upon treatment, they may recover and join recovery class *R*. Also, it is assumed that sanitation (including treatment of water bodies, safe disposal of waste) reduces pathogen concentrations in the environment. Therefore, to minimize the number of pathogens in the environment (including water sources and foods), it is essential to incorporate a rate *u*_2_(*t*) that caters for that case. Likewise, a success of education campaigns has been extensively reported by a number of scholars (e.g., [14, 16]) in combating several diseases. In the current work, we also assume that public health education plays an important role in controlling shigellosis. Education campaign is captured by a function *u*_3_(*t*). Based on these assumptions, we obtain the optimal control model:

(3) $$\begin{array}{c} \frac{dS}{dt}=\Lambda +\omega R-\left(\left(1-u_{3}\right)\lambda +\mu_{h}\right)S,\\ \frac{dE}{dt}=\left(1-u_{3}\right)\lambda S-\left(\mu_{h}+\delta \right)E,\\ \frac{dI}{dt}=q\delta E+\left(1-l\right)\alpha C-\left(\mu_{h}+d_{1}+\eta_{1}+\eta_{1}u_{1}\right)I,\\ \frac{dC}{dt}=\left(1-q\right)\delta E-\left(\mu_{h}+l\eta_{2}+\eta_{2}u_{1}+\left(1-l\right)\alpha \right)C,\\ \frac{dR}{dt}=\left(\eta_{1}+\eta_{1}u_{1}\right)I+\left(l\eta_{2}+\eta_{2}u_{1}\right)C-\left(\mu_{h}+\omega \right)R,\\ \frac{dB}{dt}=r\left(1-\frac{B}{K_{b}}\right)B+\left(1-u_{3}\right)\varepsilon_{1}I+\left(1-u_{3}\right)\varepsilon_{2}C-\left(\mu_{b}+\sigma +\sigma u_{2}\right)B, \end{array}$$

where the force of infection is

(4) $$\begin{array}{c} \lambda =\beta_{1}I+\beta_{2}C+\frac{\phi B}{K+B}, \end{array}$$

with initial conditions *S*(0) > 0; *E*(0) > 0; *I*(0) > 0; *C*(0) > 0; *R*(0) > 0; and *B*(0) > 0. It is needed to adjust these control strategies in order to minimize the number of infectious individuals and careers as well as *Shigella* bacteria and the cost of implementing the control strategies. We will consider the optimal control problem with objective functional of the form

(5) $$\begin{array}{c} J=\underset{u}{\min}\int_{0}^{t_{\text{f}}}\left(A_{1}I+A_{2}C+A_{3}B+\overset{3}{\underset{i=1}{\sum }}\frac{K_{i}}{2}u_{i}^{2}\right)dt, \end{array}$$

where *t*_f_ is the final time and *A*_*j*_, *j* = 1, 2, 3, are the weight constants associated with the number of infectious humans, carrier humans, and bacterial concentration whereas *K*_*i*_, *i* = 1, 2, 3, are the *i*^th^ weights of control relative to its cost implications. The quadratic terms (*K*_1_/2)*u*_1_^2^, (*K*_2_/2)*u*_2_^2^, and (*K*_3_/2)*u*_3_^2^ represent the costs of control efforts on treatment, sanitation, and public health education campaign, respectively. In this work, the controls *u*_*i*_, *i* = 1, 2, 3, in the objective functional are quadratic since the costs of these interventions are nonlinear. This assumption follows the works suggesting the nonlinear relationships between the effects of interventions and the cost of the intervention of the infective populations. In addition, such quadratic costs have been frequently used by several authors, for example, [18, 19]. The aim is to minimize the objective function *J*, so we are required to find the optimal control such that

(6) $$\begin{array}{c} J\left(u^{*}\right)=\min J\left(u\;|\;u\in U\right), \end{array}$$

where *U* = {(*u*_1_, *u*_2_, *u*_3_) | *u*_*i*_ is Lebesgue measurable with 0 ≤ *U* ≤ 1 for *t* ∈ [0, *t*_f_], *i* = 1, 2, 3} is the set of admissible controls. The basic setup of the optimal control problem is to check the existence and uniqueness of the optimal controls and to characterize them.

### 2.1. Existence of the Optimal Controls

In this section, we establish existence of the optimal control of the model (3) together with Equation (5) following the approach by [20] (Theorem 9.2.1 page 182). To this end, the following theorem is stated.

**Theorem 1 .**

Given that *J*(*u*) subject to system (3) with (*S*^0^, *E*^0^, *I*^0^, *C*^0^, *R*^0^, *B*^0^) ≥ (0, 0, 0, 0, 0, 0), then there exists an optimal control *u*^∗^ and corresponding (*S*^∗^, *E*^∗^, *I*^∗^, *C*^∗^, *R*^∗^, *B*^∗^) that minimizes *J*(*u*) over *U*.

**Proof**

To use the existence results from [21] (Theorem 4.1. pages 68-70), we first need to check the following properties:

1. The set of controls and corresponding state variables is nonempty
2. The measurable control set is convex and closed
3. Each right-hand side of the state system is continuous, bounded above by a sum of the bounded control and the state, and can be written as a linear function of *u* with coefficients depending on time and the state
4. The integrand *g*(*f*, *u*) of the objective functional is convex
5. There exist constants *C*_1_, *C*_2_ > 0, and *β*^∗^ ≥ 1 such that the integrand of the objective functional satisfies *g* ≥ *C*_1_(|*u*_1_|^2^ + |*u*_2_|^2^ + |*u*_3_|^2^)^*β*^∗^/2^ − *C*_2_

The existence results in [20] (Theorem 9.2.1 page 182) for the state system verify that the first property is satisfied. By definition of convex set, the control set *U* is convex and closed; hence, the second property is also satisfied. Since the state solutions of a linear state system in *u*_*i*_ are bounded, then, the right hand side is bounded by a linear function. Finally, there are *C*_1_, *C*_2_ ≥ 0 and *β* ≥ 1 satisfying *A*_1_*I* + *A*_2_*C* + *A*_3_*B* + *K*_1_*u*_1_^2^(*t*) + *K*_2_*u*_2_^2^(*t*) + *K*_3_*u*_3_^2^(*t*) ≥ *C*_1_(|*u*_1_|^2^ + |*u*_2_|^2^ + |*u*_3_|^2^)^*β*^∗^/2^ − *C*_2_ because the state variables are bounded. Hence, the existence of optimal control follows from the existence results by Fleming and Rishel [21].

### 2.2. Characterization of the Optimal Controls

The representation of the optimal controls relies on Pontryagin's Maximum Principle [17]. To apply this, we need to convert the optimal control problem into the problem of minimizing point-wise a Hamiltonian, *H*, with respect to *u*. Let *x* be the set of state variables, *U* be the set of controls, *L* be the set of adjoint variables and *f* be the right-hand side of the differential of the *i*^th^ state variable. Then, the Lagrangian function of our problem consists of the integrand of the objective functional, and the inner product of the right-hand side of the state equations and the adjoint variables (*L*_1_, *L*_2_, *L*_3_, *L*_4_, *L*_5_, *L*_6_). In more compact form, we define the Lagrangian by

(7) $$\begin{array}{c} H=A_{1}I+A_{2}C+A_{3}B+\overset{3}{\underset{i=1}{\sum }}\frac{K_{i}}{2}u_{i}^{2}+Lf\left(t,x\left(t\right),u_{i}\left(t\right)\right). \end{array}$$

The expanded form of the Lagrangian is given by

(8) $$\begin{array}{c} H=A_{1}I+A_{2}C+A_{3}B+\frac{K_{1}}{2}u_{1}^{2}+\frac{K_{2}}{2}u_{2}^{2}+\frac{K_{3}}{2}u_{3}^{2}+L_{1}\left(\Lambda +\omega R-\left(\mu_{h}+\left(1-u_{3}\right)\left(\beta_{1}I+\beta_{2}C+\frac{\phi B}{K+B}\right)\right)S\right)+L_{2}\left(\left(1-u_{3}\right)\left(\beta_{1}I+\beta_{2}C+\frac{\phi B}{K+B}\right)S-\left(\mu_{h}+\delta \right)E\right)+L_{3}\left(q\delta E+\left(1-l\right)\alpha C-\left(\mu_{h}+d_{1}+\eta_{1}+\eta_{1}u_{1}\right)I\right)+L_{4}\left(\left(1-q\right)\delta E-\left(\mu_{h}+l\eta_{2}+\eta_{2}u_{1}+\left(1-l\right)\alpha \right)C\right)+L_{5}\left(\left(\eta_{1}+\eta_{1}u_{1}\right)I+\left(l\eta_{2}+\eta_{2}u_{1}\right)C-\left(\mu_{h}+\omega \right)R\right)+L_{6}\left(r\left(1-\frac{B}{K_{b}}\right)B+\left(1-u_{3}\right)\varepsilon_{1}I+\left(1-u_{3}\right)\varepsilon_{2}C-\left(\mu_{b}+\sigma +\sigma u_{2}\right)B\right). \end{array}$$

**Theorem 2 .**

Given that *u*_*i*_^∗^ is the set of optimal control and *x*^∗^ the corresponding set of solution of the state system (3) that minimizes *J* over *Ω*, then there exist adjoint variables *L* such that

(9) $$\begin{array}{c} \frac{dL}{dt}=-\frac{dH}{dx},\text{adjoint conditions and}\\ L\left(t_{f}\right)=0,\text{transversality conditions}.\text{ Furthermore},\\ \frac{dH}{du}=0,at\;u^{*},\text{ optimality conditions}. \end{array}$$

**Proof**

The adjoint system is obtained by taking the partial derivative of the Lagrangian *H* with respect to state variables. That is,

(10) $$\begin{array}{c} \frac{dL_{1}}{dt}=\left(\mu_{h}+\left(1-u_{3}\right)\left(\beta_{1}I+\beta_{2}C+\frac{\phi B}{K+B}\right)\right)L_{1}-\left(1-u_{3}\right)\left(\beta_{1}I+\beta_{2}C+\frac{\phi B}{K+B}\right)L_{2},\\ \frac{dL_{2}}{dt}=\left(\mu +\delta \right)L_{2}-q\delta L_{3}-\left(1-q\right)\delta L_{4},\\ \frac{dL_{3}}{dt}=\left(1-u_{3}\right)\beta_{1}SL_{1}-\left(1-u_{3}\right)\beta_{1}SL_{2}+\left(\mu_{h}+d_{1}+\eta_{1}+\eta_{1}u_{1}\right)L_{3}-\left(\eta_{1}+\eta_{1}u_{1}\right)L_{5}-\left(1-u_{3}\right)\varepsilon_{1}L_{6}-A_{1},\\ \frac{dL_{4}}{dt}=\left(1-u_{3}\right)\beta_{2}SL_{1}-\left(1-u_{3}\right)\beta_{2}SL_{2}-\left(1-l\right)\alpha L_{3}+\left(\mu_{h}+l\eta_{2}+\eta_{2}u_{1}+\left(1-l\right)\alpha \right)L_{4}-\left(l\eta_{2}+\eta_{2}u_{1}\right)L_{5}-\left(1-u_{3}\right)\varepsilon_{2}L_{6}-A_{2},\\ \frac{dL_{5}}{dt}=\left(\mu_{h}+\omega \right)L_{5}-\omega L_{1},\\ \frac{dL_{6}}{dt}=\frac{\left(1-u_{3}\right)\phi KS}{{\left(K+B\right)}^{2}}\left(L_{1}-L_{2}\right)-\left(r-\mu_{b}-\sigma -\sigma u_{2}-\frac{2B}{K_{p}}\right)L_{6}-A_{3}, \end{array}$$

with transversality conditions (or final time conditions)

(11) $$\begin{array}{c} L_{1}\left(T\right)=0,\\ L_{2}\left(T\right)=0,\\ L_{3}\left(T\right)=0,\\ L_{4}\left(T\right)=0,\\ L_{5}\left(T\right)=0\\ L_{6}\left(T\right)=0. \end{array}$$

The characterizations of the optimal controls *u*^∗^(*t*) and corresponding *u*_1_^∗^(*t*), *u*_2_^∗^(*t*), *u*_3_^∗^(*t*), that is, the optimality equations, are based on the following conditions:

(12) $$\begin{array}{c} \frac{\partial H}{\partial u_{1}}=\frac{\partial H}{\partial u_{2}}=\frac{\partial H}{\partial u_{3}}=0. \end{array}$$

where

(13) $$\begin{array}{c} \frac{\partial H}{\partial u_{1}}=K_{1}u_{1}\left(t\right)-\eta_{1}IL_{3}-l\eta_{2}CL_{4}+\left(\eta_{1}I+\eta_{2}C\right)L_{5}=0,\\ \frac{\partial H}{\partial u_{2}}=K_{2}u_{2}\left(t\right)-\sigma BL_{6}=0,\\ \frac{\partial H}{\partial u_{3}}=K_{3}u_{3}\left(t\right)+\left(\beta_{1}I+\beta_{2}C+\frac{\phi B}{K+B}\right)SL_{1}-\left(\beta_{1}I+\beta_{2}C+\frac{\phi B}{K+B}\right)SL_{2}-\left(\varepsilon_{1}I+\varepsilon_{2}C\right)L_{6}=0, \end{array}$$

subject to the constraints 0 ≤ *u*_1_(*t*) ≤ *u*_1max_, 0 ≤ *u*_2_(*t*) ≤ *u*_2max_, 0 ≤ *u*_3_(*t*) ≤ *u*_3max_. Hence, on solving system (13), we have

(14) $$\begin{array}{c} u_{1}^{*}\left(t\right)=\frac{\eta_{1}L_{3}I+\eta_{2}CL_{4}-\left(\eta_{1}I+\eta_{2}C\right)L_{5}}{K_{1}},\\ u_{2}^{*}\left(t\right)=\frac{\sigma BL_{6}}{K_{2}},\\ u_{3}^{*}\left(t\right)=\frac{\left(\beta_{1}I+\beta_{2}C+\left(\phi B/K+B\right)+\mu_{h}\right)SL_{2}-\left(\beta_{1}I+\beta_{2}C+\left(\phi B/K+B\right)+\mu_{h}\right)SL_{1}+\left(\varepsilon_{1}I+\varepsilon_{2}C\right)L_{6}}{K_{3}}. \end{array}$$

Thus, using the bounds of the control *u*_1_(*t*), its optimal control is given by

(15) $$\begin{array}{c} u_{1}^{*}\left(t\right)=\left\{\begin{array}{c} \frac{\eta_{1}L_{3}I+\eta_{2}CL_{4}-\left(\eta_{1}I+\eta_{2}C\right)L_{5}}{K_{1}},\;\text{if }0\;\leq \;\frac{\eta_{1}L_{3}I+\eta_{2}CL_{4}-\left(\eta_{1}I+\eta_{2}C\right)L_{5}}{K_{1}}\leq 1,\\ 0,\;\text{if }\frac{\eta_{1}L_{3}I+\eta_{2}CL_{4}-\left(\eta_{1}I+\eta_{2}C\right)L_{5}}{K_{1}}\;\leq \;0,\\ 1,\;\text{if }\frac{\eta_{1}L_{3}I+\eta_{2}CL_{4}-\left(\eta_{1}I+\eta_{2}C\right)L_{5}}{K_{1}}\;\geq \;1. \end{array}\right. \end{array}$$

Equivalently, we can represent the optimal control as

(16) $$\begin{array}{c} u_{1}^{*}=\min\left\{1,\;\max\left\{0,\frac{\eta_{1}L_{3}I+\eta_{2}CL_{4}-\left(\eta_{1}I+\eta_{2}C\right)L_{5}}{K_{1}}\right\}\right\}. \end{array}$$

Also,

(17) $$\begin{array}{c} u_{2}^{*}\left(t\right)=\left\{\begin{array}{c} \frac{\sigma BL_{6}}{K_{2}},\;\text{if }0\;\leq \;\frac{\sigma BL_{6}}{K_{2}}\;\leq \;1,\\ 0,\;\text{if }\frac{\sigma BL_{6}}{K_{2}}\;\leq \;0,\\ 1,\;\text{if }\frac{\sigma BL_{6}}{K_{2}}\;\geq \;1. \end{array}\right. \end{array}$$

This can also be represented as

(18) $$\begin{array}{c} u_{2}^{*}=\min\left\{1,\max\left\{0,\frac{\sigma BL_{6}}{K_{2}}\right\}\right\}. \end{array}$$

Similarly,

(19) $$\begin{array}{c} u_{3}^{*}\left(t\right)=\left\{\begin{array}{c} z^{*},\;0\leq z^{*}\leq 1,\\ 0,\;\text{if }z^{*}\leq 0,\\ 1,\;\text{if }z^{*}\geq 1. \end{array}\right. \end{array}$$

where

(20) $$\begin{array}{c} z^{*}=\frac{\left(\beta_{1}I+\beta_{2}C+\left(\phi B/\left(K+B\right)\right)+\mu_{h}\right)SL_{2}-\left(\beta_{1}I+\beta_{2}C+\left(\phi B/\left(K+B\right)\right)+\mu_{h}\right)SL_{1}+\left(\varepsilon_{1}I+\varepsilon_{2}C\right)L_{6}}{K_{3}}. \end{array}$$

This can also be represented as

(21) $$\begin{array}{c} u_{3}^{*}=\min\left\{1,\max\left\{0,z^{*}\right\}\right\}. \end{array}$$

## 3. Numerical Results

In this section, the optimality system which is characterized by the state system (3), as well as the adjoint system (10), was solved numerically by using Runge-Kutta order four schemes since they provide more stable solutions as compared to the counterpart Euler's method. Euler's method is inadequate even for well-conditioned problems if a high degree of accuracy is required, owing to the slow first-order convergence. So, it is generally more convenient to use Runge-Kutta fourth-order methods. The aim was to validate the analytical results obtained in the previous sections. The implementation of the scheme was done using MATLAB package. Plots of the numerical solution are used to investigate the effect of control efforts on the population of interest.

### 3.1. Iterative Method

For a model without control, i.e., *u*_1_  =  *u*_2_  =  *u*_3_  =  0, and thus, the adjoint system does not exist, we applied a forward-in-time iterative method over the state system (1) under initial conditions *S*(0) = *S*_0_, *E*(0) = *E*_0_, *I*(0) = *I*_0_, *C*(0) = *C*_0_, *R*(0) = *R*^0^, *B*(0) = *B*_0_. However, for a model with control whose optimality conditions include a set of differential equations with initial conditions and another set with terminal conditions, we implemented the forward-backward sweep method based on the fourth-order Runge-Kutta algorithm as in [17]

- Set an initial guess for the control variables *u*_*i*_^0^(*i* = 1, 2, 3)
- Solve forward-in-time the initial value problem of the state system (3)
- Solve backwards-in-time the terminal value problem of the adjoint system (10)
- Calculate the new controls (2.13, 2.15, 2.18) with the new values of the state and adjoint solutions and then update the controls. The update of the controls can be the average between old and new controls
- Iterate the process until the solutions converge with a sufficiently small level of tolerance

### 3.2. Control Scenarios

In order to assess the impact of each control on eradication of shigellosis, the following seven control strategies were examined:

*Strategy A*: control with treatment only (*u*_1_ ≠ 0, *u*_2_ = 0, *u*_3_ = 0)

*Strategy B*: control with sanitation only (*u*_1_ = 0, *u*_2_ ≠ 0, *u*_3_ = 0)

*Strategy C*: control with education only (*u*_1_ = 0, *u*_2_ = 0, *u*_3_ ≠ 0)

*Strategy D*: control with treatment and sanitation (*u*_1_ ≠ 0, *u*_2_ ≠ 0, *u*_3_ = 0)

*Strategy E*: control with treatment and education (*u*_1_ ≠ 0, *u*_2_ = 0, *u*_3_ ≠ 0)

*Strategy F*: control with sanitation and education (*u*_1_ = 0, *u*_2_ ≠ 0, *u*_3_ ≠ 0)

*Strategy G*: control with all the three controls: treatment, sanitation, and education (*u*_1_ ≠ 0, *u*_2_ ≠ 0, *u*_3_ ≠ 0)

The parameters used for simulation are as seen in Table 8. In addition, the following initial values which were used for simulation of the optimal control are *S*(0) = 20, *E*(0) = 40, *I*(0) = 30, *C*(0) = 50, *R*(0) = 70, and *B*(0) = 90. Furthermore, the coefficients of the state and controls that were used are *A*_1_ = 0.4, *A*_2_ = 0.8, *A*_3_ = 0.3, *K*_1_ = 0.1, *K*_2_ = 0.7, and *K*_3_ = 0.5. It should be born in mind that the values of the weights used in the simulations are purely theoretical as they were arbitrarily chosen only to illustrate the control strategies proposed in this paper. Likewise, other values used for simulation are *u*_1_ = *u*_2_ = *u*_3_ = 1 and *T* = 60 days.

#### 3.2.1. Strategy A: Control with Treatment Only

**Figure 1:**
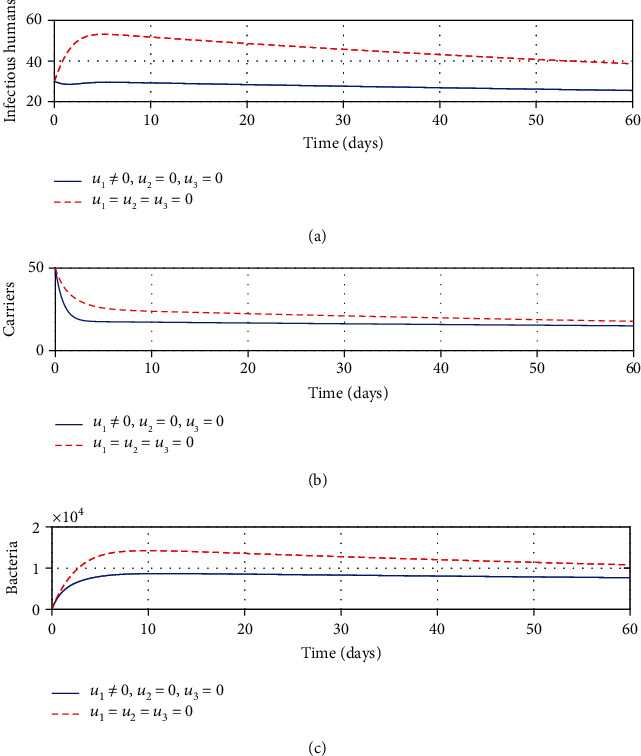
Impacts of treatment on shigellosis transmission dynamics.

We simulated the optimality system using treatment as a solely available intervention. Following the application of this strategy, it can be seen from Figure 1(a) that there is a significant decrease in the number of infectious population at a given time. A similar decline can be visualized in Figures 1(b) and 1(c) for carrier and bacterial populations, respectively. It can be noted that treatment plays a pivotal role in reducing the number of shigellosis infections. However, the results show that treatment alone is not sufficient to bring this disease to an end, thus calling for other means to work in conjuncture with treatment to contain this disease.

#### 3.2.2. Strategy B: Control with Sanitation Only

**Figure 2:**
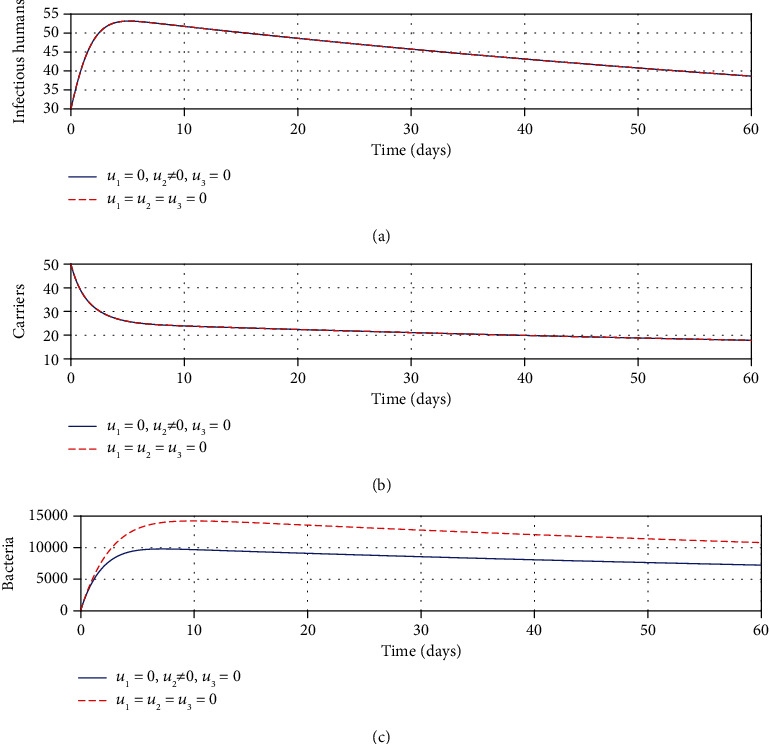
Impacts of sanitation on shigellosis transmission dynamics.

From Figures 2(a) and 2(b), it can be noted that there is no decrease in the number of infectious and carrier population, respectively, as a result of the application of sanitation. This suggests that efforts such as water chlorination and treating sewage disposal are not aimed at killing bacteria within infected individuals (*I* and *C*). However, it can be observed from Figure 2(c) that sanitation reduces the concentration of *Shigella* bacteria in the environment. This reduction might have been accelerated by sanitation activities such as water chlorination, proper sewage disposal, and high personal hygiene; all these efforts tend to limit the transmission of the epidemic shigellosis. Similarly, the results show that this strategy alone is not sufficient to eliminate the disease, especially in endemic places, thus calling for other means to work in conjuncture with sanitation to bring this disease to an end.

#### 3.2.3. Strategy C: Control with Education Only

**Figure 3:**
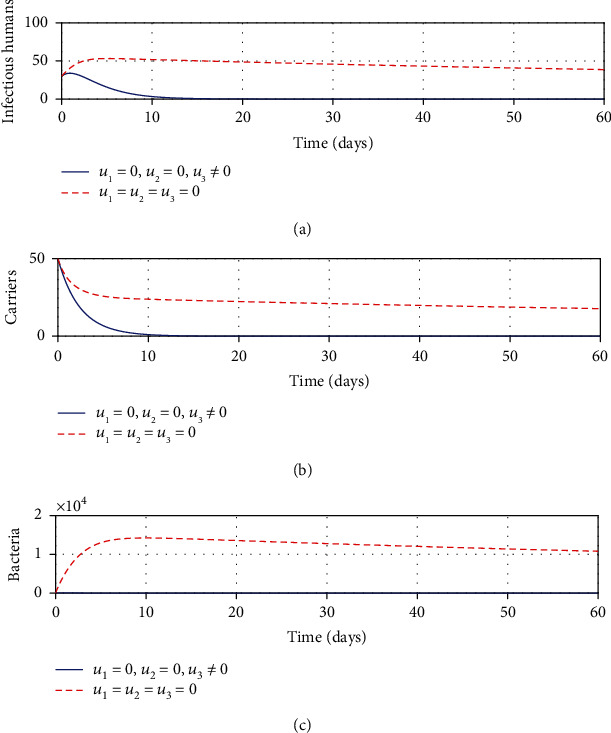
Impacts of education on shigellosis transmission dynamics.

Figures 3(a)–3(c) show that the application of this strategy yields a promising result to contain shigellosis. For example, from Figures 3(a) and 3(b) one can observe that a strict application of this strategy for a period between 10 and 20 days is enough to dwindle the number of shigellosis cases resulting from infectious as well as carrier population to zero. On the other hand, one can note in Figure 3(c) that immediate application of the same strategy from the very beginning of the control will clear the bacterial population. The finding suggests that public health education is essential to clear away the epidemic.

#### 3.2.4. Strategy D: Control with Treatment and Sanitation Only

**Figure 4:**
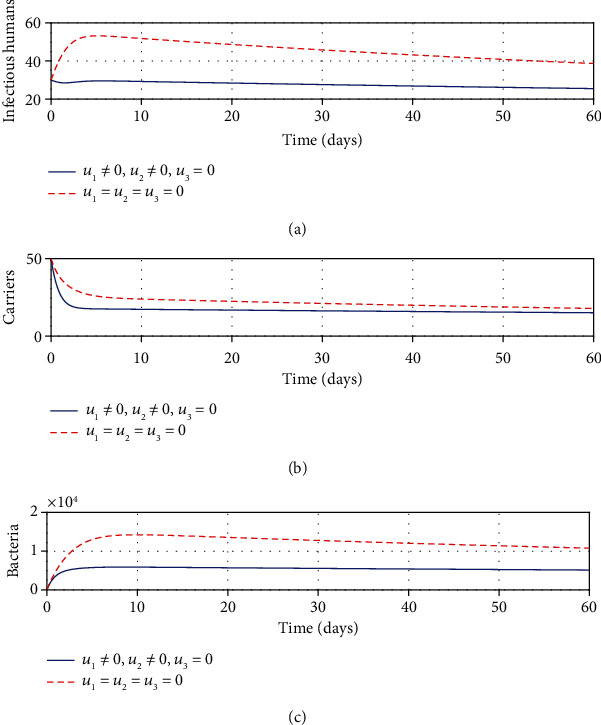
Impacts of treatment and sanitation controls on shigellosis transmission dynamics.

Figure 4(a) shows that with the application of strategy D, there is a considerable decrease in the number of infectious individuals. Likewise, Figure 4(b) shows that the carrier population decreases significantly with the application of the same strategy. Note from Figure 4(c) that the bacterial population is also affected by the implementation of this strategy. This is because, with the use of this strategy, the number of bacterial concentration tends to reduce. Even though this strategy minimizes the number of infectious, carrier, and bacterial populations, however, it seems that it is not feasible enough to eradicate shigellosis in the long run. As such, there is a need for additional control effort to curb the disease.

#### 3.2.5. Strategy E: Control with Treatment and Education Only

**Figure 5:**
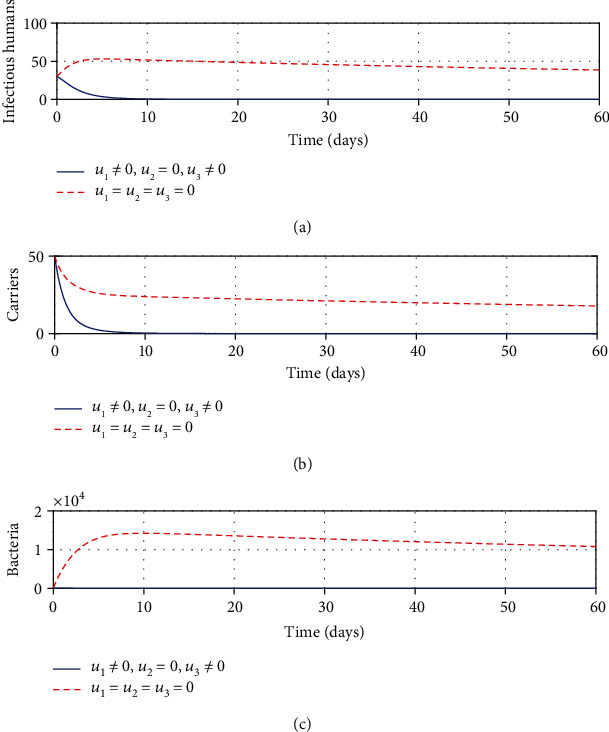
Impacts of treatment and education controls on shigellosis transmission dynamics.

Figures 5(a) and 5(b) show a sharp decrease in the number of infectious and carrier population at a given time. The disease-free state is attained earlier than 10 days of implementing this strategy. Application of the strategy is also seen as more useful to control the bacterial population from the environment(see Figure 5(c)). It can be noted that a combination of treatment and education campaign plays an important role in minimizing shigellosis infections.

#### 3.2.6. Strategy F: Control with Sanitation and Education Only

**Figure 6:**
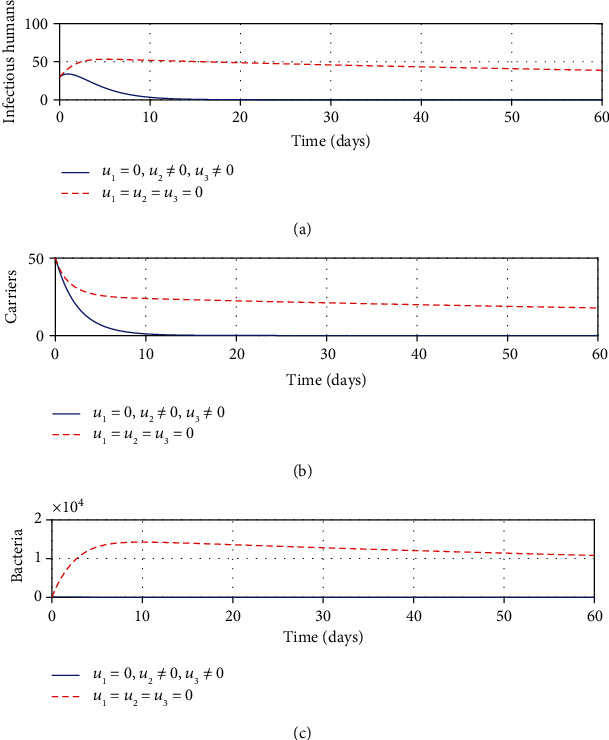
Impacts of sanitation and education controls on shigellosis transmission dynamics.

It can be seen from Figures 6(a) and 6(b) that with application of strategy F, there is a dramatic decrease in the number of infectious and carrier population at a given time. Total clearing of *Shigella* bacteria is witnessed in Figure 6(c). This implies that sanitation and education can be better used as control means of shigellosis infections.

#### 3.2.7. Strategy G: Control with Treatment, Sanitation, and Education

**Figure 7:**
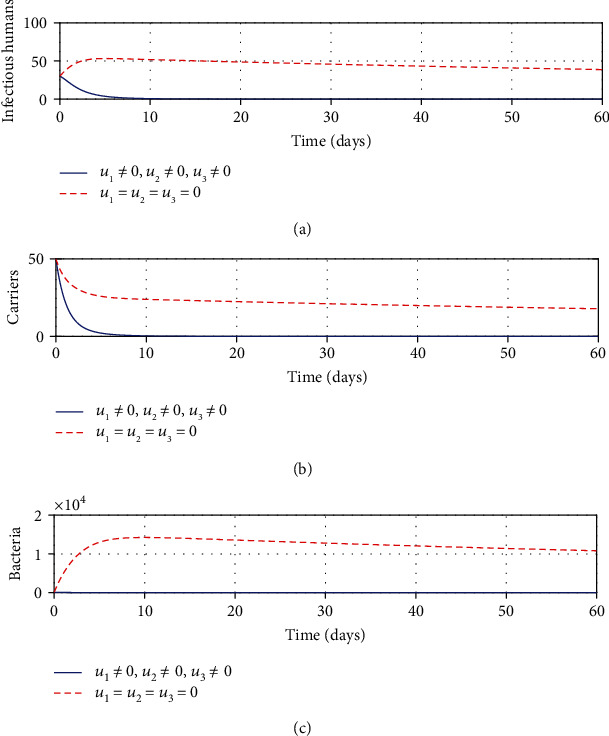
Impacts of treatment, sanitation, and education controls on shigellosis transmission dynamics.

It can be seen from Figures 7(a) and 7(b) that with the application of strategy G, there is a significant decrease in the number of infectious and carrier population at a given time. In the same vein, it can be observed from Figure 7(c) that the application of this strategy suits best to eradicate shigellosis. This result is a bit more promising than when the same controls were regarded singly or a combination of two strategies except possibly for a combination of treatment and education efforts which yield almost the same results. This result affirms the significance of applying multiple controls to contain shigellosis infections.

#### 3.2.8. Control Trajectories

**Figure 8:**
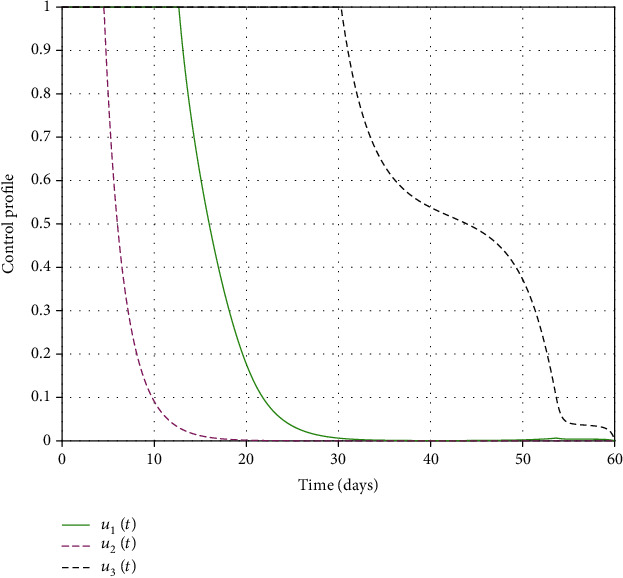
Optimal control trajectories.

It can be seen from Figure 8 that the time-dependent controls (*u*_1_, *u*_2_, *u*_3_) have also been simulated. Initially, all the time-dependent controls *u*_1_, *u*_2_, *u*_3_ are at the upper bounds, that is, *u*_1_ = *u*_2_ = *u*_3_ = 1. Each control remains constant for some time and starts to decrease gradually before it reaches the final time of application. The control *u*_1_ remains constant for about 12.66 days and becomes zero at 31.98 days, while the control *u*_2_ remains constant for about 4.38 days and becomes zero at 18.9 days, whereas the control *u*_3_ remains constant for about 30.3 days and becomes zero at 60 days. These results suggest that to prevent an outbreak, individuals in the community should continually employ treatment, sanitation effort, or education campaign at the beginning of the season. Still, as time goes on, medical doses should be minimized to reduce costs as well as its associated side effects. Sanitation of the environment should gradually decrease as well due to cost implication. However, the education campaign should be maintained at a relatively high level for the entire time of its implementation compared to other controls because its effect is evidenced for a considerable length of time.

## 4. Cost-Effectiveness Analysis

To analyze the cost-effectiveness of the strategies, we employ a more classical approach, the incremental cost-effectiveness ratio (ICER) in [22]. The ICER is applied to achieve the goal of comparing the costs and the health outcomes of two alternative intervention strategies that compete for the same resources. In ICER, when comparing two competing intervention strategies incrementally, one intervention should be compared with the next less effective alternative. It is termed as the additional cost per additional health outcome. In other words, ICER may be stated as the ratio of the difference of costs between two strategies to the difference between the total numbers of their infections averted. That is,

(22) $$\begin{array}{c} \text{ICER}\left(X\right)=\frac{\text{Cost of intervention }X-\text{cost of intervention }Y}{\text{Effect of intervention }X-\text{effect of intervention }Y}=\frac{\Delta C_{\text{T}}}{\Delta E}, \end{array}$$

where *X* and *Y* are the two intervention strategies being compared. Δ*C*_T_ is the incremental cost and Δ*E* is the incremental effect. Moreover, *C*_T_ represents the total costs incurred by implementing a particular strategy. *E* denotes the effectiveness of a specific strategy. The total number of infections averted (*E*) is estimated for each strategy by subtracting total infections with control from without control.

From this study, the total cases averted (*A*) by the intervention during the time period *t*_f_ are given by

(23) $$\begin{array}{c} A=t_{\text{f}}\left(I\left(0\right)+C\left(0\right)+B\left(0\right)\right)-\int_{0}^{t_{\text{f}}}\left(I^{*}\left(t\right)+C^{*}\left(t\right)+B^{*}\left(t\right)\right)dt, \end{array}$$

where each *I*^∗^(*t*), *C*^∗^(*t*), *B*^∗^(*t*) is the optimal solution associated with the optimal controls (*u*_1_^∗^, *u*_2_^∗^, *u*_3_^∗^) and *I*(0), *C*(0), *B*(0) is the corresponding initial condition. The initial condition is obtained as the equilibrium of system (3) with no postexposure intervention (*u*_1_ = *u*_2_ = *u*_3_ = 0), which does not depend on time, so

(24) $$\begin{array}{c} t_{\text{f}}\left(I\left(0\right)+C\left(0\right)+B\left(0\right)\right)=\int_{0}^{t_{\text{f}}}\left(I\left(0\right)+C\left(0\right)+B\left(0\right)\right)dt \end{array}$$

represents the total infectious cases over a period of *t*_f_ years.

The total cost associated with a strategy is given by

(25) $$\begin{array}{c} C_{\text{T}}=\int_{0}^{t_{\text{f}}}\left(C_{1}u_{1}\left(t\right)\left(I\left(t\right)+C\left(t\right)\right)+C_{2}u_{2}\left(t\right)B\left(t\right)+C_{3}u_{3}\left(t\right)\left(S\left(t\right)+E\left(t\right)+I\left(t\right)+C\left(t\right)\right)\right)dt, \end{array}$$

where *C*_1_ correspond to the per person unit cost following treatment intervention, *C*_2_ correspond to the per pathogen unit cost following sanitation intervention, and *C*_3_ correspond to the per person unit cost following education intervention. To proceed with ICER calculations, the alternatives that are more expensive and less ineffective are then excluded. This is done after simulating the optimal control model and then ranking strategies in order of increasing effectiveness measured as the total infections averted.

We calculate the ICER based on the strategies: A, B, C, D, E, F, and G (see details in Section 3.2). Parameter values from Table 8 are used to estimate the total cost and total infections averted that are presented in Table 1. We present some details on how to get results for Table 1. Consider strategy A, where the estimated total number of infections is 8,071,400. On the other hand, the total number of infections when there is no control strategy (status quo) was estimated to be 12,336,000. Therefore, to get the total number of averted infections for strategy A, subtract the total number of infections when there was no control strategy (*status quo*) to the total number of infections when strategy A was considered, i.e., 12,336,000 − 8,071,400 = 4,264,600. Thus, for strategy A, the number of averted infections *E* = 4,264,600. Moreover, the cost for a *status quo* strategy is $0. While, the total costs for strategy A are $50, both of them were estimated by formula (25), where *C*_1_ = 0.4, *C*_2_ = 0.8, and *C*_3_ = 0.3; in the same fashion, one can complete filling Table 1.

Table 2 incorporates ICER; it is prepared as follows: first, we rearrange control strategies from Table 1 in increasing order of effectiveness (*E*). Next, we compute incremental effectiveness Δ*E* as well as incremental costs Δ*C*_T_. The ICER is calculated by dividing incremental costs Δ*C*_T_ to incremental effectiveness Δ*E*. We calculate ICER for strategies A and B as follows:

(26) $$\begin{array}{c} \text{ICER}\left(B\right)=\frac{400880}{3909600}=0.1025,\\ \text{ICER}\left(A\right)=\frac{\left(1064.4-400880\right)}{\left(4264600-3909600\right)}=-1.1262. \end{array}$$

Comparing strategy B and strategy A, the ICER of strategy A is less than ICER of strategy B. Hence, strategy B is more costly and less effective than strategy A. Therefore, we exclude strategy B from the set of alternatives and recalculate ICER again for the remaining strategies.

Having dropped strategy B, we deduce Table 3, whose ICER are calculated as

(27) $$\begin{array}{c} \text{ICER}\left(A\right)=\frac{1064.40}{4264600}=2.4959\times 10^{-4},\\ \text{ICER}\left(D\right)=\frac{\left(262750.00-1064.40\right)}{\left(6834600-4264600\right)}=0.1018. \end{array}$$

Similarly, it is noted that the ICER of strategy A is less than ICER of strategy D. Hence, strategy D is more costly and less effective than strategy A. Therefore, we exclude strategy D from the set of alternatives and continue to compare strategies A and C.

From Table 4, we have

(28) $$\begin{array}{c} \text{ICER}\left(A\right)=\frac{1064.40}{4264600}=2.4959\times 10^{-4},\\ \text{ICER}\left(C\right)=\frac{\left(3279.3-1064.4\right)}{\left(12327018.6-4264600\right)}=2.7472\times 10^{-4}. \end{array}$$

Similarly, this comparison indicates that strategy A is cheaper than strategy C. Therefore, strategy C is rejected and continues to compare strategy A with strategy F.

From Table 5, we have

(29) $$\begin{array}{c} \text{ICER}\left(A\right)=\frac{1064.40}{4264600}=2.4959\times 10^{-4},\\ \text{ICER}\left(F\right)=\frac{\left(3212.2-1064.4\right)}{\left(12328226-4264600\right)}=2.6636\times 10^{-4}. \end{array}$$

Similarly, this comparison indicates that strategy A is cheaper than strategy F. Therefore, strategy F is rejected and continues to compare strategy A with strategy E.

From Table 6, we have

(30) $$\begin{array}{c} \text{ICER}\left(A\right)=\frac{1064.40}{4264600}=2.4959\times 10^{-4},\\ \text{ICER}\left(E\right)=\frac{\left(3852.6-1064.4\right)}{\left(12329683.5-4264600\right)}=3.4572\times 10^{-4}. \end{array}$$

Again, the comparison indicates that strategy A is cheaper than strategy E. Therefore, strategy E is ignored and continues to compare strategy A with the last strategy, which is G. From Table 7, we have

(31) $$\begin{array}{c} \text{ICER}\left(A\right)=\frac{1064.40}{4264600}=2.4959\times 10^{-4},\\ \text{ICER}\left(G\right)=\frac{\left(2914.6-1064.4\right)}{\left(12330926-4264600\right)}=2.2937\times 10^{-4}. \end{array}$$

Finally, the comparison result reveals that strategy G is cheaper than strategy A. Therefore, strategy G (treatment, education, and sanitation) is the best of all possible strategies due to its cost-effectiveness and healthy benefits.

## 5. Conclusion

In this study, a basic model that traces the evolution of shigellosis is developed and presented; an optimal control problem has been obtained by modifying the basic model. We have established the existence of an optimal control problem and later analyzed the full optimal control system. We have solved the optimality system numerically and established its findings. The findings from optimal control show that the strategy that includes all three controls (treatment, sanitation, and education) plays a crucial role in diminishing the outbreak. Similarly, it was observed that any strategy under consideration that incorporated public health education seemed more beneficial than the one that ignored it. Moreover, we have assessed the cost-effectiveness of the control strategies established using the ICER method and noted that the most cost-effective strategy was the one that incorporates all three control efforts (treatment, sanitation, and education).

## Figures and Tables

**Table 1:** Number of infections averted and total cost of each strategy.

Strategies	Infections	Infection averted (*E*)	Costs ($) (*C*_T_)
*Status quo*	12,336,000	—	0
A	8,071,400	4,264,600	1064.4
B	8,426,400	3,909,600	400,880
C	8981.4	12,327,018.6	3279.3
D	5,501,400	6,834,600	262,750
E	6316.5	12,329,683.5	3852.6
F	7774	12,328,226	3212.2
G	5074	12,330,926	2914.6

**Table 2:** Incremental cost-effectiveness ratios of different optimal control strategies.

Strategies	*E*	Δ*E*	*C*_T_	Δ*C*_T_	ICER (Δ*C*_T_/Δ*E*)
B	3,909,600	3,909,600	400,880	400,880	0.1025
A	4,264,600	355,000	1064.4	-399,815.6	-1.1262
D	6,834,600	2,570,000	262,750	261,685.6	0.1018
C	12,327,018.6	5,492,418.6	3279.3	-259,470.7	−4.7242 × 10^−2^
F	12,328,226	1207.4	3212.2	-67.1	−5.5574 × 10^−2^
E	12,329,683.5	1457.5	3852.6	640.4	0.4394
G	12,330,926	1242.5	2914.6	-938	-0.7549

**Table 3:** Incremental cost-effectiveness ratios of different optimal control strategies excluding strategy B.

Strategies	*E*	Δ*E*	*C*_T_	Δ*C*_T_	ICER (Δ*C*_T_/Δ*E*)
A	4,264,600	4,264,600	1064.4	1064.4	2.4959 × 10^−4^
D	6,834,600	2,570,000	262,750	261,685.6	0.1018
C	12,327,018.6	5,492,418.6	3279.3	-259,470.7	−4.7242 × 10^−2^
F	12,328,226	1207.4	3212.2	-67.1	−5.5574 × 10^−2^
E	12,329,683.5	1457.5	3852.6	640.4	0.4394
G	12,330,926	1242.5	2914.6	-938	-0.7549

**Table 4:** Incremental cost-effectiveness ratios of different optimal control strategies excluding strategies B and D.

Strategies	*E*	Δ*E*	*C*_T_	Δ*C*_T_	ICER (Δ*C*_T_/Δ*E*)
A	4,264,600	4,264,600	1064.4	1064.4	2.4959 × 10^−4^
C	12,327,018.6	8,062,418.6	3279.3	2214.9	2.7472 × 10^−4^
F	12,328,226	1207.4	3212.2	-67.1	−5.5574 × 10^−2^
E	12,329,683.5	1457.5	3852.6	640.4	0.4394
G	12,330,926	1242.5	2914.6	-938	-0.7549

**Table 5:** Incremental cost-effectiveness ratios for optimal control strategies A, E, F, and G.

Strategies	*E*	Δ*E*	*C*_T_	Δ*C*_T_	ICER (Δ*C*_T_/Δ*E*)
A	4,264,600	4,264,600	1064.4	1064.4	2.4959 × 10^−4^
F	12,328,226	8,063,626	3212.2	2147.8	2.6636 × 10^−4^
E	12,329,683.5	1457.5	3852.6	640.4	0.4394
G	12,330,926	1242.5	2914.6	-938	-0.7549

**Table 6:** Incremental cost-effectiveness ratios for optimal control strategies A, E, and G.

Strategies	*E*	Δ*E*	*C*_T_	Δ*C*_T_	ICER (Δ*C*_T_/Δ*E*)
A	4,264,600	4,264,600	1064.4	1064.4	2.4959 × 10^−4^
E	12,329,683.5	8,065,083.5	3852.6	2788.2	3.4572 × 10^−4^
G	12,330,926	1242.5	2914.6	-938	-0.7549

**Table 7:** Incremental cost-effectiveness ratios for optimal control strategies A and G.

Strategies	*E*	Δ*E*	*C*_T_	Δ*C*_T_	ICER (Δ*C*_T_/Δ*E*)
A	4,264,600	4,264,600	1064.4	1064.4	2.4959 × 10^−4^
G	12,330,926	8,066,326	2914.6	1850.2	2.2937 × 10^−4^

## Data Availability

The data used to support the findings of this study are included within the article.

## Supplementary Materials

Supplementary Materials Table 8: parameters and their description. Table 9: state variables and their description [23–27].
