# Supplementary material for: Optimal Control of Shigellosis with Cost-Effective Strategies
Source: Comput Math Methods Med. 2020 Aug 28;2020:9732687. doi: 10.1155/2020/9732687 (PMC7474761; doi:10.1155/2020/9732687)
Supplement: Supplementary Materials — Table 8: parameters and their description. Table 9: state variables and their description [23–27]. [file 9732687.f1.zip › TABLE_ 8 _Parameters_Descrition_Values.pdf]

Table 8: Parameters and their description.

**Parameter**	**Description**	**Value**	**Source**
Λ	Rate of recruitment of individuals into the susceptible class	462 Humans/year	[10]
β_1	Transmission rate for infectious individuals	0.4/year	[10]
β_2	Transmission rates for carriers	0.6/year	[10]
ϕ	Effective transmission rate of *Shigellosis* due to environment to human interaction	0.4465/year	[23]
δ	Incubation rate (Rate at which exposed individuals, $E(t)$ progress to either class $I(t)$ or $C(t)$)	0.35/day	[27]
μ_h	Natural human mortality rate	0.4465/year	Assumed
q	A fraction of exposed individuals, $E(t)$ who progress to class $I(t)$	0.9	[8]
l	A fraction of carriers, $C(t)$ who recover naturally and progress to class $R(t)$	0.4	Assumed
α	Rate of screening carriers	0.56	Assumed
d_1	Disease induced death rate by $I(t)$	0.02 /year	Assumed
η_1	Recovery rate of infectious humans	0.14/day	[8]
η_2	Recovery rate of carrier humans	0.0286/day	[8]
K_p	The environmental carrying capacity for *Shigella* bacteria in the food or water supply	10^4 cells/mL	Assumed
K	Half saturation rate of *Shigella* bacteria that can cause a 50% chance of infection	60 cells/mL	Assumed
ϵ_1	Bacteria shed rate into the water supply by infectious human	80 cells/mL/day	[26]
ϵ_2	Bacteria shed rate into the water supply by carrier humans human	70 cells/mL/day	[26]
r	(Maximum) per capita growth rate of *Shigella* bacteria	0.73/day	[25]
μ_b	Mortality rate of *Shigella* bacteria, including phage degradation	0.83/day	Assumed
ω	Wanning rate of diseased induced immunity	0.25 /year	[8]
σ	Rate at which sanitation leads to death of *Shigella* bacteria	1.66/day	[24]
γ	Treatment rate for infectious individuals(I)	0.4/year	Assumed
ρ	Education efficacy parameter	0.6/year	[25]
