# Supplementary material for: Optimal Control of Shigellosis with Cost-Effective Strategies
Source: Comput Math Methods Med. 2020 Aug 28;2020:9732687. doi: 10.1155/2020/9732687 (PMC7474761; doi:10.1155/2020/9732687)
Supplement: Supplementary Materials — Table 8: parameters and their description. Table 9: state variables and their description [23–27]. [file 9732687.f1.zip › TABLE_9 _State Variables_Description.pdf]

Table 9: State variables and their description.

**Variable**	**Description**
$S(t)$	The number of susceptible humans at a time t.
$E(t)$	The number of exposed humans at a time t.
$I(t)$	The number of infectious humans at a time t.
$R(t)$	The number of recovered (immune) humans at a time t.
$C(t)$	The number of carriers at a time t.
$B(t)$	The number of *Shigella* bacteria at a time t.
$\lambda_h(t)$	Human-to-human transmission at time t.
$\lambda_h(t)$	Environment-to-human transmission at time t.
